# The non-homologous end-joining activity is required for Fanconi anemia fetal HSC maintenance

**DOI:** 10.1186/s13287-019-1206-0

**Published:** 2019-03-29

**Authors:** Yan Nie, Yibo Li, Xiaoli Li, Andrew F. Wilson, Qishen Pang

**Affiliations:** 1 Division of Experimental Hematology and Cancer Biology, Cincinnati Children’s Hospital Medical Center, 3333 Burnet Avenue, Cincinnati, OH 45229 USA; 2 Department of Pediatrics, University of Cincinnati College of Medicine, Cincinnati, OH 45229 USA

**Keywords:** Fanconi anemia (FA), Hematopoietic stem cells (HSCs), Homologous recombination (HR), Non-homologous end joining (NHEJ)

## Abstract

**Background:**

Recent studies have shown that deficiency in the Fanconi anemia (FA) DNA repair pathway enhances the error-prone non-homologous end-joining (NHEJ) repair, leading to increased genomic instability, and that genetic or pharmacological inhibition of the NHEJ pathway could rescue the FA phenotype.

**Methods:**

First, we exposed LSK cells from WT and *Fanca*^*−/−*^ mice to DNA-PKcs inhibitor NU7026 or Ku70 knockdown to examine whether inhibition of NHEJ sensitizes *Fanca*^*−/−*^ HSPCs to PARP inhibitor (PARPi)- or interstrand crosslinking (ICL)-induced cell death and genomic instability. We then generated *DNA-PKcs*^*3A/3A*^*Fanca*^*−/−*^ mice to investigate the effect of specific inactivation of NHEJ on fetal HSCs. Lastly, we used two p53 mutant models to test whether specific inactivation of the p53 function in apoptosis is sufficient to rescue embryonic lethality and fetal HSC depletion in *Fanca*^*−/−*^ *DNA-PKcs*^*3A/3A*^ mice.

**Results:**

Inhibition of NHEJ sensitizes HSPCs from *Fanca*^*−/−*^ mice to PARP inhibition- and ICL-induced cell death and genomic instability and further decreases *Fanca*^*−/−*^ HSPC proliferation and hematopoietic repopulation in irradiated transplant recipients. Specific inactivation of NHEJ activity by the knockin *DNA-PKcs*^*3A/3A*^ mutation in two FA mouse models, *Fanca*^*−/−*^ and *Fancc*^*−/−*^, leads to embryonic lethality. *DNA-PKcs*^*3A/3A*^ causes fetal HSC depletion in developing *Fanca*^*−/−*^ embryos due to increased HSC apoptosis and cycling. Both p53^−/−^ and a knockin *p53*^*515C*^ mutation, which selectively impairs the p53 function in apoptosis, can rescue embryonic lethality and fetal HSC depletion in *Fanca*^*−/−*^ *DNA-PKcs*^*3A/3A*^ mice.

**Conclusion:**

These results demonstrate that the NHEJ pathway functions to maintain Fanconi anemia fetal HSCs.

## Background

Fanconi anemia (FA) is a genetic disorder associated with bone marrow (BM) failure and malignancies including leukemia and solid cancers [1–4]. Mutations in any of the 22 FA genes (*FANCA-W*) lead to clinical manifestations characterized by developmental abnormalities, progressive bone marrow failure (BMF), and a high risk of developing cancer including leukemia [5–8]. At the cellular level, FA is characterized by chromosomal instability and DNA cross-linker sensitivity, which serves as a clinical diagnostic hallmark of FA [1–4]. At the molecular level, eight FA proteins (FANCA, -B, -C, -E, -F, -G, -L, and -M), along with other associated factors, form the FA core complex in response to DNA damage or replicative stress, which acts in part as an ubiquitin ligase. This FA core complex monoubiquitinates two downstream FA proteins, FANCD2 and FANCI, which then recruit other downstream FA proteins including several key proteins involved in homologous recombination (HR) repair, and possibly other DNA repair factors, to nuclear loci containing damaged DNA and consequently influence important cellular processes such as DNA replication, cell-cycle control, and DNA damage response and repair [9–11].

Recent studies suggested that the FA pathway promotes the error-free HR repair pathway while suppressing the error-prone non-homologous end-joining (NHEJ) pathway [12–15]. Using FA-deficient *Caenorhabditis* *elegans*, chicken and human cells, two studies demonstrated that FA deficiency enhanced the error-prone NHEJ repair, leading to increased genomic instability [12, 15]. These studies also showed that genetic or pharmacological inhibition of the NHEJ pathway could rescue the FA phenotype. Another similar study showed that inhibition of the NHEJ ligase, LIG4, ameliorated the FA phenotype, but had no effect on BRCA1 deficiency [16]. It appears the FA pathway may act to prevent inappropriate recruitment of NHEJ factors to sites of DNA damage. However, the exact mechanism by which the FA pathway counteracts the NHEJ pathway is largely unknown.

A clinical application of HR-NHEJ interaction is synthetic lethality induced by poly (ADP-ribose) polymerase (PARP) inhibition in *BRCA1/2*-mutated cancer [17, 18]. Since PARP functions as a critical sensor of single-strand breaks (SSBs) in base-excision repair, as a mediator for restarting stalled replication forks of HR-mediated double-strand break (DSB) repair, and as a means of preventing the binding of Ku proteins to DNA ends in NHEJ pathway [19–22], therefore, blocking the ADP-ribosylation activity with small molecules can achieve synthetic lethality with DNA-damaging agents in the treatment of certain cancers [23–29]. It has been shown that PARP inhibitors could selectively target cancer cells with a defective HR repair of DSB [25]. For example, *BRCA1*-, *BRCA2*-, and *ATM*-deficient cells show hypersensitivity to PARP inhibitors, leading to genomic instability and eventual cell death due to the development of non-viable genetic errors generated by the error-prone NHEJ repair [26–28].

In the current study, we show that inhibition of NHEJ sensitizes *Fanca*^*−/−*^ HSPCs from mice to PARP inhibition-induced cell death and genomic instability and leads to a further decrease in the proliferation and hematopoietic repopulation of the *Fanca*^*−/−*^ HSPCs. We also show that simultaneous inactivation of DNA-PKcs and Fanca or Fancc causes embryonic lethality in mice, which can be rescued by the apoptosis-defective p53 mutation. Furthermore, using the knockin *DNA-PKcs*^*3A/3A*^ model, which specifically inactivates the NHEJ activity of DNA-PKcs, we demonstrate that the NHEJ activity of DAN-PKcs is required for FA fetal HSC maintenance.

## Methods

### Mice and treatment

*Fanca*^*−/−*^ and *Fancc*^*−/−*^ mice [30, 31] were generated by interbreeding the heterozygous *Fanca*^*+/−*^ (Dr. Madeleine Carreau at Laval University) or *Fancc*^*+/−*^ mice (Dr. Manuel Buchwald, University of Toronto), respectively. *p53*^*515C/515C*^ mice (provided by Dr. Guillermina Lozano at University of Texas M.D. Anderson Cancer Center) [32] or *DNA-PKcs*^*3A/3A*^ mice (provided by Dr. Benjamin P. C. Chen at University of Texas Southwestern Medical Center) [33] were generated by interbreeding heterozygous *p53*^*+/515C*^ or *DNA-PKcs*^+/*3A*^ mice, respectively. All the animals including BoyJ mice were maintained in the animal barrier facility at Cincinnati Children’s Hospital Medical Center. All animal experiments were performed in accordance with the institutional guidelines and approved by the Institutional Animal Care and Use Committee of Cincinnati Children’s Hospital Medical Center (IACUC2018-0006).

### Isolation of bone marrow cells and flow cytometry analysis

The femora and tibiae were harvested from the mice immediately after their sacrifice with CO_2_. Bone marrow (BM) cells were flushed from bones into Iscove’s modified Dulbecco’s medium (IMDM; Invitrogen) containing 10% FCS, using a 21-gauge needle and syringe. Low-density BM mononuclear cells (LDBMMNCs) were separated by Ficoll Hypaque density gradient (Sigma-Aldrich, St. Louis, MO) and washed with IMDM medium.

For flow analysis and cell sorting, the lineage marker (Lin) mixture (BD Biosciences, San Jose, CA) for BM cells from treated or untreated mice included the following biotinylated antibodies: CD3ε (145-2C11), CD11b (M1/70), CD45R/B220 (RA3-6B2), and mouse erythroid cells Ly-76 (Ter119), Ly6G, and Ly-6C (RB6-8C5). Other conjugated antibodies (BD Biosciences, San Jose, CA) used for surface staining included CD45.1 (A20), CD45.2 (A104), Sca1 (D7), c-kit (2B8), CD48 (HM48-1), and CD150 (9D1). Biotinylated primary antibodies were detected by incubation of antibody-coated cells with streptavidin-PerCP or FITC (BD Biosciences, San Jose, CA) in a two-step staining procedure. For the detection of fetal liver HSCs, whole fetal liver cells were incubated with FITC-conjugated antibody to CD41 (MWReg30), CD48 (HM48-1-PE), Ter119 (Ter119), PE-conjugated antibody to CD150 (26D12:DNAX), APC-conjugated Mac1 (M1/70), and biotin-conjugated Sca1 (Ly6A/E-biotin), followed by staining with streptavidin conjugated to APC-Cy7 (PharRed, PR; Becton Dickinson). For BM transplantation experiments, pacific blue-conjugated CD45.2 (A104, BioLegend, San Diego, CA) was used to determine donor-derived cells. For cell sorting, lineage-negative cells were enriched using lineage depletion reagents (StemCell Technologies) according to the manufacturer’s instruction. The Lin-negative and LSK populations were acquired by using the FACSAria II sorter (BD Biosciences).

### In vitro cell culture and treatment

Briefly, LSK cells were maintained in StemSpan medium supplemented with 50 ng/ml murine rTpo (Preprotech, Rocky Hill, NJ), 50 ng/ml murine rSCF (Preprotech, Rocky Hill, NJ), and 1% BSA at 37 °C in normoxia (21% O_2_, 5% CO_2_). Cells with the indicated genotype were treated with increasing doses of DNA-PKcs inhibitor NU7026 (0–100 μM; Sigma-Aldrich, St Louis, MO), PARP inhibitor KU58948 (1 μM; Axon Medchem), or mitomycin C (0–1.0 μM; Sigma-Aldrich, St Louis, MO) for 36 h followed by survival and chromosomal breakage analyses.

### Ku70 knockdown by lentiviral short hairpin RNA

Hairpin sequence for scramble control (CTCGCTTGGGCGAGAGTAA) or *Ku70-1* (CCCAGAGTGTGTACACCAGTAA), *Ku70-2* (CCGTCAGATTGTGCTGGAGAAA), and *Ku70-3* (ACGACACAGGTGGAGAATATAA) was cloned into SFLV-eGFP-shRNA vector (Dr. Lenhand Rudolph (Institute of Molecular Medicine and Max-Planck-Research, Germany). The plasmids (10 μg each) were used to produce retroviral supernatant. LSK cells were transduced with the lentiviral supernatants in various volumes (5, 10, 20, 40, and 80 μL). Protein was harvested 48 h after transduction and used for Western blot analysis of Ku-70 using anti-Ku70 mouse monoclonal antibody (mab-Ku70, 3114-500, Abcam).

### Chromosomal breakage analysis

Chromosome breakage analysis was performed on LSK cells as previously described [34]. Briefly, cells were treated with 0.05 mg/ml colcermid (Gibco, Grand Island, NY, USA) for 90 min, followed by 0.4% KCl hypotonic solution at 37° for 20 min, fixed with methanol and acetic acid at 4° for 15 min, and dropped onto microscope slides. The cells were then rinsed with isoton, stained with Giemsa for 5 min, and rinsed with Gurr Buffer (CTL Scientific, Deer Park, NY, USA) and Milli-Q-filtered deionized water. A total of 50 cells from each sample were scored for chromosome aberrations.

### Bone marrow transplantation (BMT)

One thousand to 2000 LSK cells (CD45.2^+^), along with 200,000 c-Kit-depleted protector cells, were transplanted into lethally irradiated BoyJ (CD45.1^+^) mice. The recipients were subjected to flow cytometric analysis for donor-derived LSK cells 16 weeks after BMT. In other experiments, 2000 GFP-sorted scramble shRNA or *Ku70* shRNA lentiviral vector-transduced LSK cells, along with 200,000 c-Kit-depleted protector cells, were transplanted into lethally irradiated BoyJ mice. The recipients were subjected to flow cytometric analysis for donor-derived LSK cells 16 weeks after BMT.

### Cell-cycle and apoptosis analysis

To analyze the cell-cycle status of the HSC subsets, bone marrow cells were initially stained with antibodies against Lin^+^ cells, C-KIT, SCA-1, CD150, and CD48 as described above. After incubation with these cell surface antibodies, the cells underwent fixation and permeabilization with transcription factor buffer set (BD Biosciences, #562725) according to the manufacturer’s instruction. After fixation, cells were incubated with APC-anti-Ki67 (BD Biosciences, #558615), washed and stained with PI. Cells were analyzed by flow cytometry. For the apoptosis detection, bone marrow cells were stained with the antibodies for the HSC surface markers and then stained with APC-Annexin V (BD Biosciences, #550474) and 7 AAD. Annexin V-positive populations were determined as apoptotic cells using the FACS LSR II (BD Biosciences).

### Colony-forming unit assay

For the in vitro colony-forming unit (CFU) assay, 1000 sorted LSK cells were seeded in MethoCult GF M3434 (STEMCELL Technologies) according to the manufacturer’s recommendations. Colonies were visualized and counted at day 7. The experiment was performed in triplicate for each sample.

### Statistical analysis

Student’s *t* test was performed using GraphPad Prism v6 (GraphPad software). Comparison of more than two groups was analyzed by one-way ANOVA test. Values of *p* < 0.05 were considered statistically significant. Results are presented as mean ± SD. “*” indicates *p* < 0.05; “**”, *p* < 0.01; and “***”, *p* < 0.001.

## Results

### Inhibition of NHEJ sensitizes *Fanca*^*−/−*^ HSPCs to PARPi-induced cell death and genomic instability

**Fig. 1:**
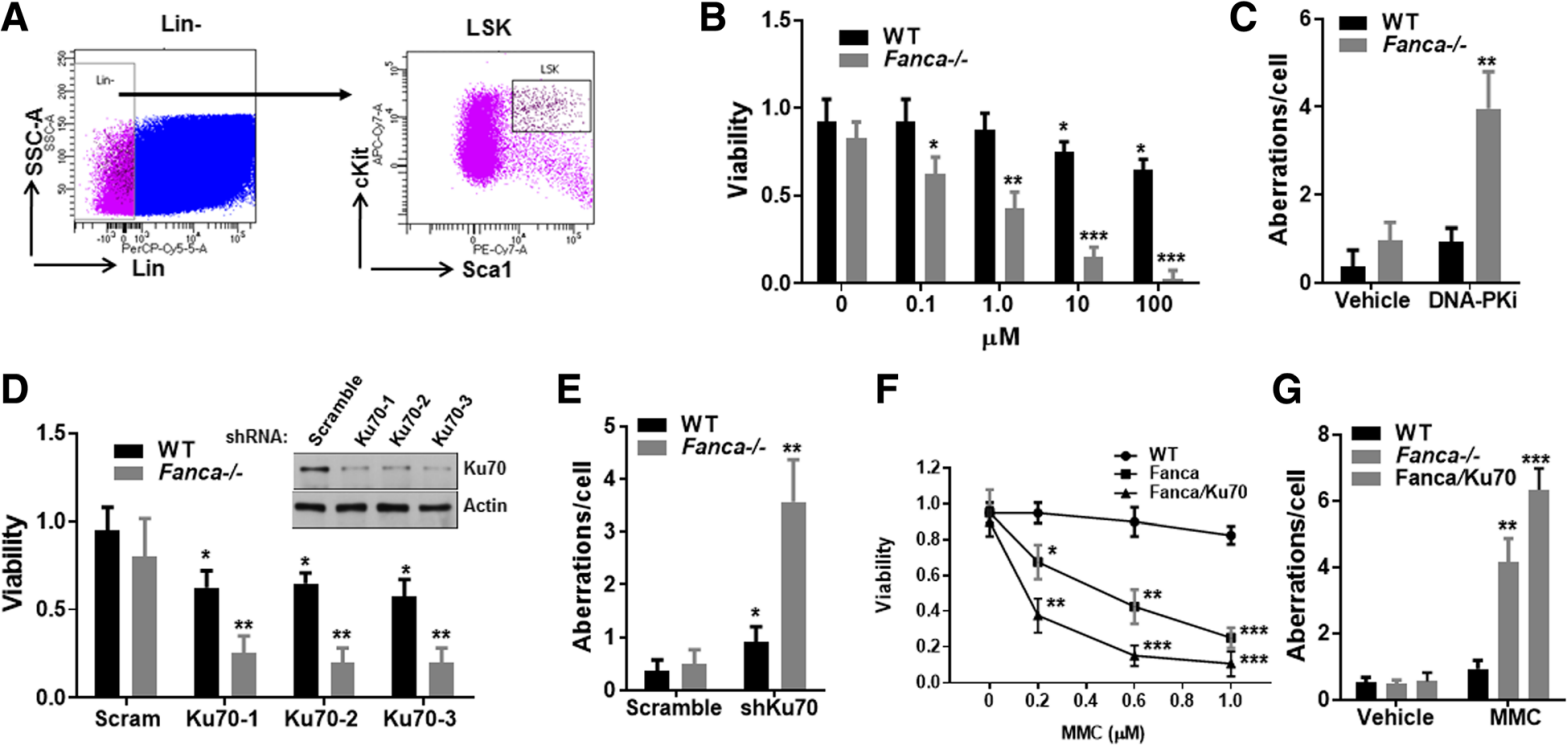
Inhibition of NHEJ sensitizes *Fanca*^*−/−*^ HSPCs to PARPi-induced cell death and genomic instability. **a** Gating strategy for sorting HSPCs (Lin^−^Sca1^+^c-kit^+^; LSK). **b** Inhibition of DNA-PKcs further sensitizes *Fanca*^*−/−*^ HSPCs to PARPi-induced cell death. BM LSK cells isolated from wild-type (WT) or *Fanca*^*−/−*^ mice were treated with increasing doses of DNA-PKcs inhibitor NU7026 in the presence of PARP inhibitor KU58948 (1 μM; Axon Medchem) for 36 h. Cell viability was determined by trypan blue assay. Percentages of viable cells were normalized to that of WT control at dose 0 μM. *p < 0.05, **p < 0.01, or ***p < 0.001 vs WT control at dose 0 μM. **c** Inhibition of DNA-PKcs exacerbates genomic instability in *Fanca*^*−/−*^ LSK cells. BM LSK cells isolated from WT or *Fanca*^*−/−*^ mice were treated with DNA-PKcs inhibitor NU7026 (10 μM), or vehicle control, in the presence of PARP inhibitor KU58948 (1 μM) for 36 h. The cells were subjected to chromosomal breakage analysis. Quantification of chromosomal aberrations in 50 cells in random fields is shown. **p < 0.01 vs WT vehicle control. **d** Knockdown of Ku70 increases cell death in *Fanca*^*−/−*^ HSPCs. BM LSK cells from WT or *Fanca*^*−/−*^ mice were transduced with lentiviruses co-expressing eGFP and scramble shRNA or shRNA targeting *Ku70*. Transduced cells were sorted for eGFP expression and treated with PARP inhibitor KU58948 (1 μM) for 36 h. Cell viability was determined by trypan blue assay. Percentages of viable cells were normalized to that of WT cells transduced with the scramble shRNA control. Insert: Ku70 expression in cells expressing Ku70 shRNAs. *p < 0.05 or **p < 0.01 vs WT scramble shRNA control. **e** Knockdown of Ku70 exacerbates genomic instability in *Fanca*^*−/−*^ LSK cells. The cells described in **c** were subjected to chromosomal breakage analysis. Quantification of chromosomal aberrations in 50 cells in random fields is shown. *p < 0.05 or **p < 0.01 vs WT scramble shRNA control. **f** Knockdown of Ku70 increases MMC-induced cell death in *Fanca*^*−/−*^ HSPCs. WT and *Fanca*^*−/−*^ or *Fanca*^*−/−*^ LSK cells expressing Ku70 shRNA (*Fanca*^*−/−*^/Ku70) were treated with increasing doses of MMC (0–1.0 μM) for 36 h. Cell viability was determined by trypan blue assay. Percentages of viable cells were normalized to that of WT cells transduced with the scramble shRNA control. *p < 0.05, **p < 0.01, or **p < 0.001 vs WT scramble shRNA control. **g** Knockdown of Ku70 exacerbates MMC-induced genomic instability in *Fanca*^*−/−*^ HSPCs. The cells described in **f** were treated with MMC (0.2 μM) for 36 h and then subjected to chromosomal breakage analysis. Quantification of chromosomal aberrations in 50 cells in random fields is shown. **p < 0.01 or ***p < 0.001 vs WT scramble shRNA control

To understand the mechanism by which the FA pathway counteracts NHEJ in genomic maintenance in HSPCs, we exposed BM LSK (Lin^−^Sca1^+^c-kit^+^; Fig. 1a) cells from WT and *Fanca*^*−/−*^ mice to DNA-PKcs inhibitor NU7026 or Ku70 knockdown in the presence of PARP inhibitor KU58948. The reason for PARP inhibition was that we and others have shown that PARP inhibition could greatly boost NHEJ activity in HR-deficient cells including FA HSPCs [28, 29, 35]. Both WT and *Fanca*^*−/−*^ LSK cells were not sensitive to the PARP inhibitor (Fig. 1b). However, treatment with the DNA-PKcs inhibitor NU7026 sensitized the *Fanca*^*−/−*^ LSK cells to PARPi-induced cell death at low doses (0.1–1 μM), which had no effect on WT cells (Fig. 1b). Furthermore, inhibition of DNA-PKcs exacerbated genomic instability (chromosome and chromatid breaks, and radial chromosomes) in *Fanca*^*−/−*^ LSK cells (Fig. 1c). We also genetically inhibited NHEJ by knocking down Ku70 expression using lentiviral shRNAs (Fig. 1d). We found that knockdown of Ku70 caused much higher levels of cell death (Fig. 1d) and chromosome aberrations (Fig. 1e) in *Fanca*^*−/−*^ LSK cells than in WT cells. Furthermore, we treated BM LSK cells from WT and *Fanca*^*−/−*^ mice with DNA cross-linker mitomycin C (MMC), which induces interstrand crosslinking (ICL), and found that knockdown of Ku70 caused much higher levels of cell death (Fig. 1d) and chromosome aberrations (Fig. 1e) in *Fanca*^*−/−*^ LSK cells compared to *Fanca*^*−/−*^ mock control cells. Together, these results suggest that the NHEJ pathway actually contributes to cell survival and genomic maintenance in *Fanca*^*−/−*^ HSPCs.

### Inhibition of NHEJ further decreases *Fanca*^*−/−*^ HSPC renewal and repopulation

**Fig. 2:**
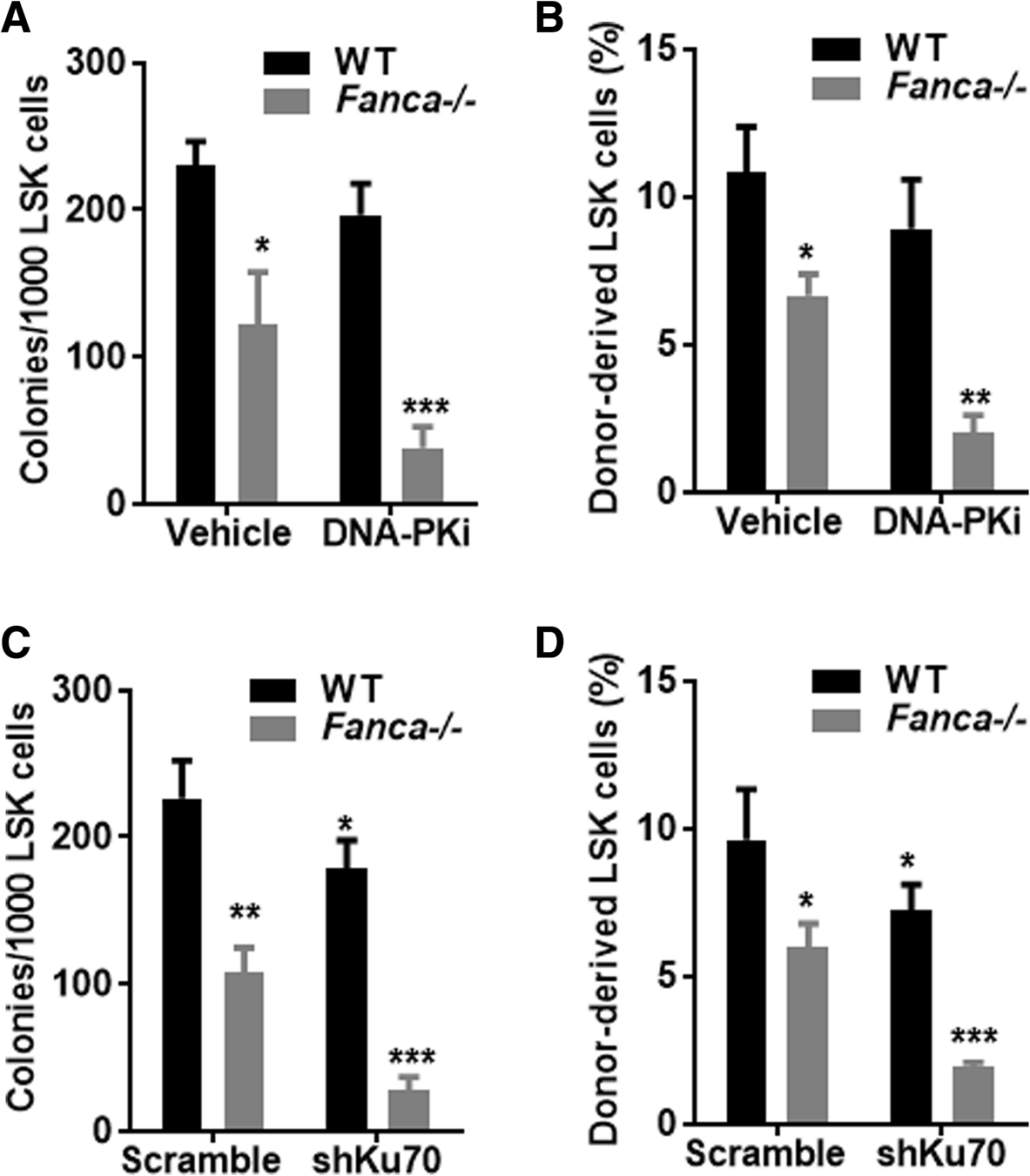
Inhibition of NHEJ further decreases *Fanca*^*−/−*^ HSC renewal and repopulation. **a** Inhibition of DNA-PKcs further decreases *Fanca*^*−/−*^ HSPC proliferation. BM LSK cells isolated from WT or *Fanca*^*−/−*^ mice were treated with DNA-PKcs inhibitor NU7026 (10 μM) for 36 h and then plated in cytokine-supplemented methylcellulose medium. Colonies were enumerated on day 7 after plating. Results are means ± standard deviation (SD) of three independent experiments. *p < 0.05 or ***p < 0.001 vs WT vehicle control. **b** Inhibition of DNA-PKcs further compromises the repopulating capacity of *Fanca*^*−/−*^ HSPCs. BM LSK cells isolated from WT or *Fanca*^*−/−*^ mice were treated with DNA-PKcs inhibitor NU7026 (10 μM) for 36 h. Two thousand LSK cells, along with 200,000 c-Kit-depleted protector cells, were then transplanted into lethally irradiated BoyJ mice. The recipients were subjected to flow cytometric analysis for donor-derived LSK cells 16 weeks after BMT (*n* = 9–12 per group). *p < 0.05 or **p < 0.01 vs WT vehicle control. **c** Knockdown of Ku70 further decreases *Fanca*^*−/−*^ HSPC proliferation. BM LSK cells from WT or *Fanca*^*−/−*^ mice were transduced with lentiviruses co-expressing eGFP and scramble shRNA or shRNA targeting *Ku70*. Transduced cells were sorted for eGFP expression and then plated in cytokine-supplemented methylcellulose medium. Colonies were enumerated on day 7 after plating. Results are means ± standard deviation (SD) of three independent experiments. *p < 0.05, **p < 0.01, or ***p < 0.001 vs WT scramble shRNA control. **d** Knockdown of Ku70 compromises the repopulating capacity of *Fanca*^*−/−*^ HSPCs. Two thousand LSK cells described in **c**, along with 200,000 c-Kit-depleted protector cells, were then transplanted into lethally irradiated BoyJ mice. The recipients were subjected to flow cytometric analysis for donor-derived LSK cells 16 weeks after BMT (*n* = 9 per group). *p < 0.05 or ***p < 0.001 vs WT scramble shRNA control

We next determined the effect of NHEJ inhibition on the proliferation of *Fanca*^*−/−*^ HSPCs using the in vitro colony-forming unit (CFU) assay and the in vivo hematopoietic repopulation assay. Inhibition of NHEJ by the DNA-PKcs inhibitor NU7026 further reduced the capacity of *Fanca*^*−/−*^ LSK cells to produced colony formation units when plated in methylcellulose supplemented with hematopoietic cytokines (Fig. 2a) and decreased the potential of these cells to proliferate in irradiated transplant recipients (Fig. 2b). Similar results were obtained with the *Fanca*^*−/−*^ LSK cells that had been subjected to knockdown of Ku70 (Fig. 2c, d). Specifically, knocking down Ku70 further compromised the ability of *Fanca*^*−/−*^ LSK cells to form colony in the absence of stromal support (Fig. 2c) and to repopulate the transplant recipient mice (Fig. 2d). Taken together, these results indicate a crucial role of NHEJ in maintaining *Fanca*^*−/−*^ HSPC proliferation.

### Inactivation of the NHEJ activity of DNA-PKcs in *Fanca*^*−/−*^ or *Fancc*^*−/−*^ mice leads to embryonic lethality

The observation that inhibition of NHEJ exacerbated genomic instability in *Fanca*^*−/−*^ HSPCs appears to be conflict with previous reports that inhibition of the key NHEJ factors such as Ku, Lig4, or DNA-PKcs could ameliorate the sensitivity of FA cells to interstrand crosslinking agents [12, 15]. This prompted us to determine the in vivo effect of NHEJ inhibition in *Fanca*^*−/−*^ mice. We crossed the *Fanca*^*−/−*^ mice with a strain carrying the knockin *DNA-PKcs*^*3A/3A*^ mutation, which selectively inactivates the NHEJ activity but does not affect the kinase activity of DNA-PKcs [33]. To exclude the probability that the identified phenotypes might be due to a specific effect of a particular FA complementation group, we also employed an additional FA (*Fancc*^*−/−*^) mouse model. Screening more than 160 E10.5 embryos and 270 pups showed that while we were able to obtain *DNA-PKcs*^*+/3A*^*Fanca*^*−/−*^ and *DNA-PKcs*^*+/3A*^*Fancc*^*−/−*^ pups, we found that *DNA-PKcs*^*3A/3A*^*Fanca*^*−/−*^ or *DNA-PKcs*^*3A/3A*^*Fancc*^*−/−*^ double-deficient mice did not survive to birth (Tables 1 and 2). Thus, these results indicate that simultaneous inactivation of *DNA-PKcs* and *Fanca* or *Fancc* causes embryonic lethality in mice.

**Table 1:** Survival of *Fanca*^*−/−*^ *DNA-PKcs*^*3A/3A*^ embryos and pups

*DNA-PKcs*^*+/3A*^ *Fanca*^*+/−*^ *× DNA-PKcs*^*+/3A*^ *Fanca*^*+/−*^ intercross					
		*DNA-PKcs*^*3A/3A*^ *Fanca*^*+/+*^ or *DNA-PKcs*^*3A/3A*^ *Fanca*^*+/−*^	*DNA-PKcs*^*+/+*^ *Fanca*^*−/−*^ or *DNA-PKcs*^*+/3A*^ *Fanca*^*−/−*^	*DNA-PKcs* ^*3A/3A*^ *Fanca* ^*−/−*^	Other genotypes
E10.5 embryos (96 screened)	Expected	24	24	6	42
	Observed	22	21	5	48
Live pups (170 screened)	Expected	42	42	10	76
	Observed	35	36	0	99

**Table 2:** Survival of *Fancc*^*−/−*^ *DNA-PKcs*^*3A/3A*^ embryos and pups

*DNA-PKcs*^*+/3A*^ *Fancc*^*+/−*^ *× DNA-PKcs*^*+/3A*^ *Fancc*^*+/−*^ intercross					
		*DNA-PKcs*^*3A/3A*^ *Fancc*^*+/+*^ or *DNA-PKcs*^*3A/3A*^ *Fancc*^*+/−*^	*DNA-PKcs*^*+/+*^ *Fancc*^*−/−*^ or *DNA-PKcs*^*+/3A*^ *Fancc*^*−/−*^	*DNA-PKcs* ^*3A/3A*^ *Fancc* ^*−/−*^	Other genotypes
E10.5 embryos (56 screened)	Expected	14	14	3	25
	Observed	12	13	2	29
Live pups (106 screened)	Expected	26	26	6	48
	Observed	19	22	0	65

### *DNA-PKcs*^*3A/3A*^ causes fetal HSC depletion in *Fanca*^*−/−*^ embryos due to increased HSC apoptosis and cycling

**Fig. 3:**
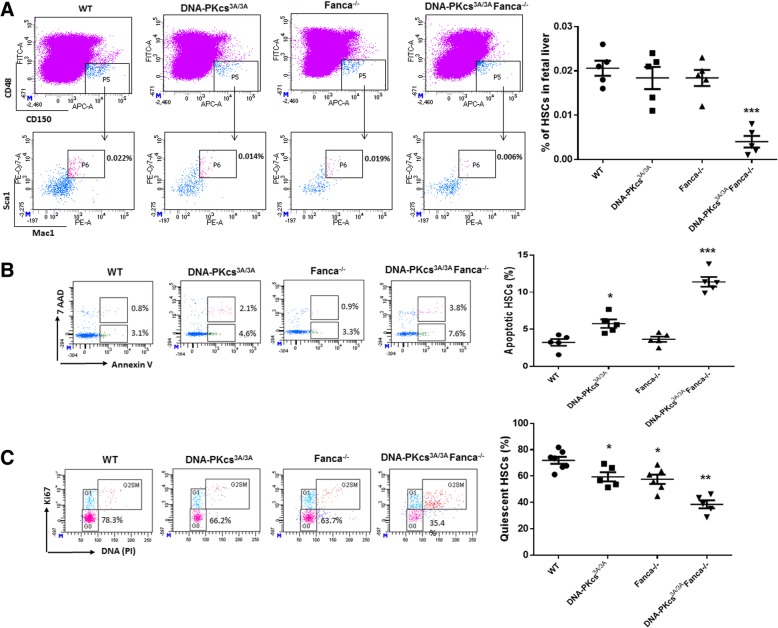
Inhibition of NHEJ causes fetal HSC depletion in *Fanca*^*−/−*^ embryos. **a** *DNA-PKcs*^*3A/3A*^ induces fetal HSC depletion in *Fanca*^*−/−*^ embryos. Fetal liver cells from E14.5 embryos with the indicated genotype were subjected to flow cytometric analysis for fetal HSC (CD150^+^CD48^−^Lin^−^Mac-1^+^Sca-1^+^). Representative flow cytometric plots (left) and quantification (right) are shown. ***p < 0.001 vs WT control. **b** Increased apoptosis in *DNA-PKcs*^*3A/3A*^ *Fanca*^*−/−*^ fetal HSCs. Fetal HSCs from E14.5 embryos of the indicated genotype were analyzed for apoptosis by Annexin V and 7AAD. Representative flow cytometric plots (left) and quantification (right) are shown. *p < 0.05 or ***p < 0.001 vs WT control. **c** Decreased quiescence in *DNA-PKcs*^*3A/3A*^ *Fanca*^*−/−*^ fetal HSCs. The percentage of quiescent (G_0_) fetal HSCs in E14.5 embryos of the indicated genotype. Representative flow cytometric plots (left) and quantification (right) are shown. *p < 0.05 or **p < 0.01 vs WT control

We next investigated the effect of *DNA-PKcs-Fanca* deficiencies on fetal hematopoiesis by examining the frequency of fetal HSCs (CD150^+^CD48^−^Lin^−^Mac-1^+^Sca-1^+^) in the E14.5 fetal liver of the mice, which has been shown to include all fetal liver HSC activity and are highly enriched for HSCs [36]. As shown in Fig. 3a, the frequency of fetal HSCs was more than four- to fivefold lower in *DNA-PKcs*^*3A/3A*^*Fanca*^*−/−*^ fetal livers compared to control samples from WT or single-deficient (*Fanca*^*−/−*^ or *DNA-PKcs*^*3A/3A*^) mice (Fig. 3a), indicating a phenotype of fetal HSC depletion.

Because we observed exacerbated cell death in *Fanca*^*−/−*^ LSK cells upon NHEJ inhibition (Fig. 1b, d), we wondered if increased apoptosis played a causal role in the depletion of fetal HSCs in *DNA-PKcs*^*3A/3A*^*Fanca*^*−/−*^ mice. To examine this possibility, we measured the apoptosis of fetal liver cells in WT, *Fanca*^*−/−*^, *DNA-PKcs*^*3A/3A*^, and *DNA-PKcs*^*3A/3A*^*Fanca*^*−/−*^ embryos at E14.5 by Annexin V staining. Low levels (approximately 5%) of apoptotic cells were observed in the livers of both WT and *Fanca*^*−/−*^ embryos (Fig. 3b). Whereas there was a significant increase in apoptotic fetal HSCs in *DNA-PKcs*^*3A/3A*^ embryos compared to WT and *Fanca*^*−/−*^ embryos, this increase was greatly exacerbated in *DNA-PKcs*^*3A/3A*^*Fanca*^*−/−*^ fetal livers (Fig. 3b). These results suggest that fetal HSC depletion observed in *DNA-PKcs*^*3A/3A*^*Fanca*^*−/−*^ mice may be caused by increased apoptosis. We also performed cell-cycle analysis to evaluate the effect of *DNA-PKcs*^*3A/3A*^ on quiescence of *Fanca*^*−/−*^ fetal HSCs. We observed a statistically significant reduction of quiescent fetal HSCs in *DNA-PKcs*^*3A/3A*^ and *Fanca*^*−/−*^ embryos compared with WT embryos (Fig. 3c). Interestingly, a more dramatic decrease in quiescent fetal HSCs was detected in *DNA-PKcs*^*3A/3A*^*Fanca*^*−/−*^ embryos compared with the other three groups (Fig. 3c). These results suggest that the NHEJ activity of DNA-PKcs and Fanca may play a quantitative or collaborative functional role in the cell cycle of fetal HSCs.

### Inactivation of the p53 function in apoptosis is sufficient to rescue embryonic lethality and fetal HSC depletion in *Fanca*^*−/−*^*DNA-PKcs*^*3A/3A*^ mice

**Fig. 4:**
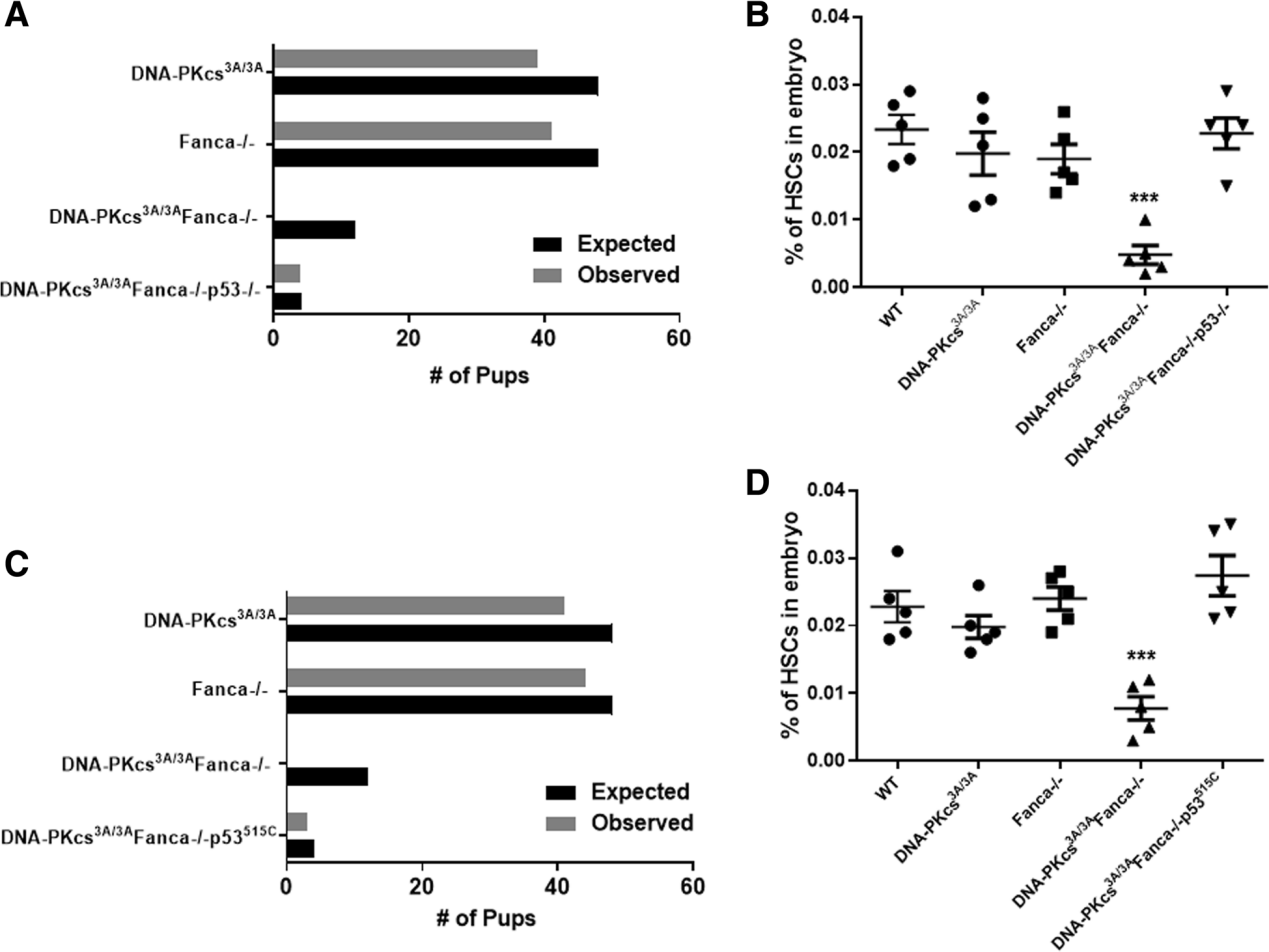
Inactivation of p53 apoptosis function rescues embryonic lethality and fetal HSC depletion in *Fanca*^*−/−*^ *DNA-PKcs*^*3A/3A*^ mice. **a** Deletion of p53 rescues embryonic lethality and fetal HSC depletion in *Fanca*^*−/−*^ *DNA-PKcs*^*3A/3A*^ mice. Graphical representation of expected vs. observed number of pups based on Mendelian inheritance of alleles. **b** Deletion of p53 rescues fetal HSC depletion in *Fanca*^*−/−*^ *DNA-PKcs*^*3A/3A*^ mice. Fetal liver cells from E14.5 embryos with the indicated genotype were subjected to flow cytometric analysis for fetal HSC (CD150^+^CD48^−^Lin^−^Mac-1^+^Sca-1^+^). ***p < 0.001 vs WT control. **c** *p53*^*515C*^ rescues embryonic lethality in *Fanca*^*−/−*^ *DNA-PKcs*^*3A/3A*^ mice. Graphical representation of expected vs. observed number of pups based on Mendelian inheritance of alleles. **d** *p53*^*515*^ rescues fetal HSC depletion in *Fanca*^*−/−*^ *DNA-PKcs*^*3A/3A*^ mice. Fetal liver cells from E14.5 embryos with the indicated genotype were subjected to flow cytometric analysis for fetal HSC (CD150^+^CD48^−^Lin^−^Mac-1^+^Sca-1^+^). ***p < 0.001 vs WT control

Elevated p53 activation has been reported in the *DNA-PKcs*^*3A/3A*^ HSCs and FA HSPCs [33, 37]. We thus asked whether p53-dependent apoptosis played a role in embryonic lethality and fetal HSC depletion in *Fanca*^*−/−*^*DNA-PKcs*^*3A/3A*^ mice. To this end, we bred *Fanca*^*+/−*^*DNA-PKcs*^*+/3A*^ mice to *p53*^*−/−*^ animals and assessed the viability and development of the fetal HSCs. Because we observed increased HSC cycling in *DNA-PKcs*^*3A/3A*^*Fanca*^*−/−*^ embryos (Fig. 3c), we also crossed *Fanca*^*+/−*^*DNA-PKcs*^*+/3A*^ mice to a mutant *p53* mouse strain harboring a separation-of-function mutation in p53, *p53*^*515C*^, in which its apoptotic function is abolished but its cell-cycle checkpoint activities remain intact [32]. The viability of *DNA-PKcs*^*3A/3A*^*Fanca*^*−/−*^ mice was rescued by both *p53*-null deficiency and the *p53*^*515C*^ allele Fig. 4a, c). Furthermore, both the *p53*-null and the *p53*^*515C*^ allele were able to rescue fetal HSC depletion in *Fanca*^*−/−*^*DNA-PKcs*^*3A/3A*^ embryos (Fig. 4b, d). Therefore, the p53-dependent apoptosis plays a causal role in embryonic lethality and fetal HSC depletion in *Fanca*^*−/−*^*DNA-PKcs*^*3A/3A*^ mice.

## Discussion

In the present study, we used multiple mouse models of closely related DNA damage response (FA, NHEJ, p53) pathways to show that inhibition of NHEJ sensitizes *Fanca*^*−/−*^ HSPCs to PARPi-induced cell death and genomic instability. This surprising finding prompted us to propose that inhibition of the NHEJ pathway in FA HSPCs might actually exacerbate their sensitivity to DNA damage, which is the cellular hallmark of FA. In support of this notion, we showed that specific inactivation of the NHEJ activity of DNA-PKcs caused embryonic lethality in mice deficient for two components of the FA core complex *Fanca* and *Fancc*. Our results are in strike contrast to the studies reported by Adamo et al. [12] and Pace et al. [15] that hypersensitivity of human, nematode, and chicken DT40 cells to interstrand crosslinking agents can be rescued by knockdown, deletion, or inhibition of major NHEJ proteins such as Ku, Lig4, or DNA-PKcs. The discrepancy between these studies and ours may be due to the difference in species and cell types that were used in the experiments. It is noteworthy that the human cell lines and the chicken DT40 cells employed in the previous studies are known to utilize the HR pathway for DSB repair; whereas HSPCs in our study use the NHEJ pathway predominantly for repair of DSBs [38]. Interestingly, a more recent study shows that deletion of *Ku80*, another NHEJ factor, also causes embryonic lethality in mice deficient for *Fancd2* [39].

The cause of embryonic lethality in *DNA-PKcs*^*3A/3A*^*Fanca*^*−/−*^ mice may be due to fetal HSC depletion. In support of this notion, we observed significantly increased HSC apoptosis and cycling in developing embryos of *DNA-PKcs*^*3A/3A*^*Fanca*^*−/−*^ mice compared to those of WT, *DNA-PKcs*^*3A/3A*^ or *Fanca*^*−/−*^ mice. It is well known that aberrantly increased cell cycling can lead to the depletion of adult HSCs, which are quiescent under normal conditions [40–42]. Our results raise the possibility that abnormally increased cell-cycle progression in fetal HSCs could also lead to their depletion. Interestingly, both p53 null and a knockin *p53*^*515C*^ mutation, which selectively impairs only the p53 function in apoptosis, can rescue embryonic lethality and fetal HSC depletion in *Fanca*^*−/−*^ *DNA-PKcs*^*3A/3A*^ mice. This suggests that although *DNA-PKcs*^*3A/3A*^ increases *Fanca*^*−/−*^ HSC cycling, the cell-cycle activity of p53 is not the decisive factor in the regulation of *DNA-PKcs*^*3A/3A*^ HSC maintenance. In this context, our results are consistent with previous studies that show p53-dependent apoptosis in the *DNA-PKcs*^*3A/3A*^ HSCs and FA HSPCs [33, 37].

## Conclusions

In this study, we employed multiple mouse models of closely related DNA damage response (FA, NHEJ, p53) pathways to demonstrate that the NHEJ pathway is required for cell survival and proliferation of murine FA HSPCs. We further show that the NHEJ pathway functions to maintain Fanconi anemia fetal HSCs.
